# The pH-Dependent Controlled Release of Encapsulated Vitamin B_1_ from Liposomal Nanocarrier

**DOI:** 10.3390/ijms22189851

**Published:** 2021-09-12

**Authors:** Ádám Juhász, Ditta Ungor, Egon Z. Várkonyi, Norbert Varga, Edit Csapó

**Affiliations:** 1 MTA-SZTE “Momentum” Noble Metal Nanostructures Research Group, Interdisciplinary Excellence Center, Department of Physical Chemistry and Materials Science, University of Szeged, Rerrich B. Sqr. 1, H-6720 Szeged, Hungary; juhaszad@chem.u-szeged.hu (Á.J.); ungord@chem.u-szeged.hu (D.U.); varkonyizegon@gmail.com (E.Z.V.); varganorbi0000@gmail.com (N.V.); 2 MTA-SZTE Biomimetic Systems Research Group, Department of Medical Chemistry, University of Szeged, Dóm Sqr. 8, H-6720 Szeged, Hungary

**Keywords:** asolectin, liposome, vitamin B_1_, nanocarrier, encapsulation, pH-controlled release

## Abstract

In this work, we firstly presented a simple encapsulation method to prepare thiamine hydrochloride (vitamin B_1_)-loaded asolectin-based liposomes with average hydrodynamic diameter of ca. 225 and 245 nm under physiological and acidic conditions, respectively. In addition to the optimization of the sonication and magnetic stirring times used for size regulation, the effect of the concentrations of both asolectin carrier and initial vitamin B_1_ on the entrapment efficiency (EE %) was also investigated. Thermoanalytical measurements clearly demonstrated that after the successful encapsulation, only weak interactions were discovered between the carriers and the drug molecules. Moreover, the dissolution profiles under physiological (pH = 7.40) and gastric conditions (pH = 1.50) were also registered and the release profiles of our liposomal B_1_ system were compared with the dissolution profile of the pure drug solution and a manufactured tablet containing thiamin hydrochloride as active ingredient. The release curves were evaluated by nonlinear fitting of six different kinetic models. The best goodness of fit, where the correlation coefficients in the case of all three systems were larger than 0.98, was reached by application of the well-known second-order kinetic model. Based on the evaluation, it was estimated that our liposomal nanocarrier system shows 4.5-fold and 1.5-fold larger drug retention compared to the unpackaged vitamin B_1_ under physiological conditions and in artificial gastric juice, respectively.

## 1. Introduction

In the last decades, one of the major challenges of medicine has been the development of novel, more effective drug carrier systems with biocompatible features [1]. The advantages of colloidal carriers in contrast to traditional formulations lie in their cellular effect, at which nearly 100% of the active ingredients can enter the cell and thus their bioavailability can be significantly increased. Besides the surface modification and selective functionalization of the colloidal particle, the targeted delivery of the chosen drugs can also be ensured. Thanks to these benefits, the side effects of the drug molecules can be reduced, and the dose can be increased at the same time. Although many publications can be found which discuss the benefits of the inorganic-based drug delivery systems [2,3,4], these carriers are difficult to remove from the human body [5]. Recently, the development of organic-based nanocarriers has been the focus of extensive research thanks to their outstanding biocompatible and biodegradable properties [1]. Besides biocompatible polymers [6,7,8,9,10], serum albumins [11,12,13] can be also used to synthesize several biocompatible drug delivery systems applying the layer-by-layer (LbL) preparation technique. The liposomes (LIPs) are also potential carriers due to the amphiphilic properties of the phospholipid building blocks [14]. They have a similar chemical structure to the cell membrane, which is conducive for the easier endocytosis of the drugs. These spherical hollow particles are composed of various phospholipid bilayers, where the hydrophilic part is orientated towards the aqueous medium, while the hydrophobic part is located in the interior of the double layer. With the compact structure, LIPs are also suitable for encapsulating water-soluble and non-water-soluble molecules into the internal aqueous core or into the double lipid layer, respectively. In addition to being used prominently in cancer therapy [15,16,17,18], it should be also noted that it is one of the defining nanostructures of our decade due to its major role in the defense against the COVID-19 pandemic as nanocarriers for the vaccination and sensor measurements [19].

At present, perhaps in addition to vitamin C, there are relatively few commercially available liposomal-based vitamin-containing medicinal products in which the active ingredient is exclusively in a liposome-stored form. In the presented work, vitamin B_1_ was selected for the development of a liposomal-based colloidal drug carrier. Vitamin B_1_ is one of the most important vitamins for the human body; its absence can lead to beriberi disease caused by impaired glucose oxidation and lack of pyruvate oxidation [20]. This essential compound also acts as a coenzyme of the enzyme that decarboxylates red tartaric acid. The accumulation of red tartaric acid and lactic acid can cause nervousness, anorexia, circulatory failure, and heart failure [21,22]. Considering these, it can produce feelings of weakness and fatigue, depression, and intestinal problems in some cases. These symptoms can be detected in those people who have deficient or poor nutrition or alcoholic addiction [23,24].

The aim of this work is the optimization of the encapsulation protocols of thiamine hydrochloride in different media. To the best of our knowledge, S. J. Fathima et al. have attempted to make liposomal thiamine dietary supplements [25] using lecithin and *_L_*-α-phosphatidylcholine so far. The multilayered LIPs were characterized by light scattering, calorimetry, and atomic force microscopy. Nevertheless, the exact measurement of the drug release, as well as the kinetic evaluation of the dissolution profiles, were neglected, which are outstandingly important key questions for future applications. In contrast, the development of a liposomal-based colloidal carrier was carried out in this work by a selection of more cost-effective asolectin, which is a natural phospholipid and fatty acid mixture from soybeans. The effects of several parameters (such as sonication time, the concentration of the lipid, and drug content) on the entrapment efficiency (EE %) were investigated. Furthermore, the analyses of the in vitro drug dissolution profiles under physiological conditions (phosphate buffer, pH = 7.4 and 0.15 M NaCl) and in artificial gastric juice (pH = 1.5 and 0.2 M KCl) were also highlighted. The results of the presented work can help to develop LIP-based oral and intravenous products for delivery of the selected essential vitamin B_1_ drug molecule. As can be seen in the following, both the optimization protocols were worked out, and both the release profiles were registered and analyzed under physiological and acidic conditions for this purpose.

## 2. Results and Discussion

### 2.1. Optimization of the Drug Encapsulation in Different Media

To facilitate the development of a possible production method for a liposomal-based thiamine (vitamin B_1_)-containing delivery system, two pH conditions were selected for preparation. For future intravenous application, a physiological condition was applied firstly, which was imitated by phosphate buffer solution (PBS, pH = 7.4 with 0.15 M NaCl). Based on the different colloid stability of the drug-free asolectin-based LIPs (Figure S1), the acidic condition (pH = 3.0) was ensured by 0.001 M HCl solution containing 0.2 M KCl, which served as a good model for an oral liposome formulation of the selected vitamin. It is well known that the encapsulation protocols, as well as the size-controlling process, have a great effect on the EE %. For the preparation, a simple one-pot in situ method was chosen to fill the LIPs with vitamin B_1,_ where the uniform lipid film was rehydrated in the aqueous solution of thiamine hydrochloride using 1 mM concentration of this vitamin. This can be defined as an in situ encapsulation protocol which easily entraps a large amount of drug molecule into the LIPs. The final lipid concentration was 1 mg/mL. To adjust the smallest sizes associated with an appropriate EE %, the widely used ultrasound treatment was applied.

**Figure 1:**
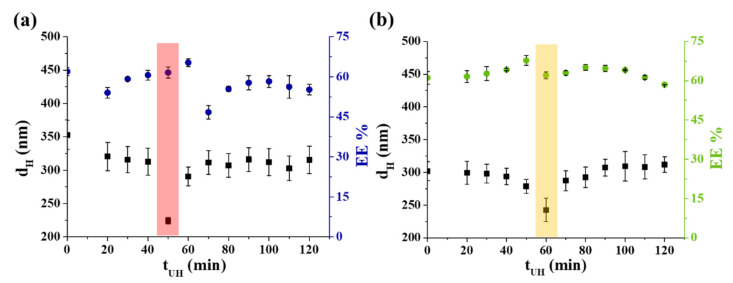
The hydrodynamic diameter (d_H_) and the EE % of the vitamin B_1_-containing LIPs depending on the sonication time (t_UH_) (**a**) at pH = 7.40 and (**b**) at pH = 3.00. In both cases the optimal treatment times are highlighted (colored columns).

To find the optimal sonication time after the 10 min magnetic stirring of the components, the hydrodynamic diameters (d_H_) of the LIPs were registered using dynamic light scattering (DLS) and the vitamin B_1_ content was identified by spectrophotometry at the same time under both physiological and acidic conditions. As can be seen in Figure 1, the optimal sonication time is ca. 50 min in PBS solution (Figure 1a), resulting in the average diameter of d_H_ = 224.2 ± 5.1 nm with EE % = 61.5 ± 2.3%. In contrast, at acidic condition (pH = 3.00), 60 min sonication provides 242.6 ± 17.8 nm average size for the formed LIPs, while the EE % is 62.0 ± 1.31%.

**Figure 2:**
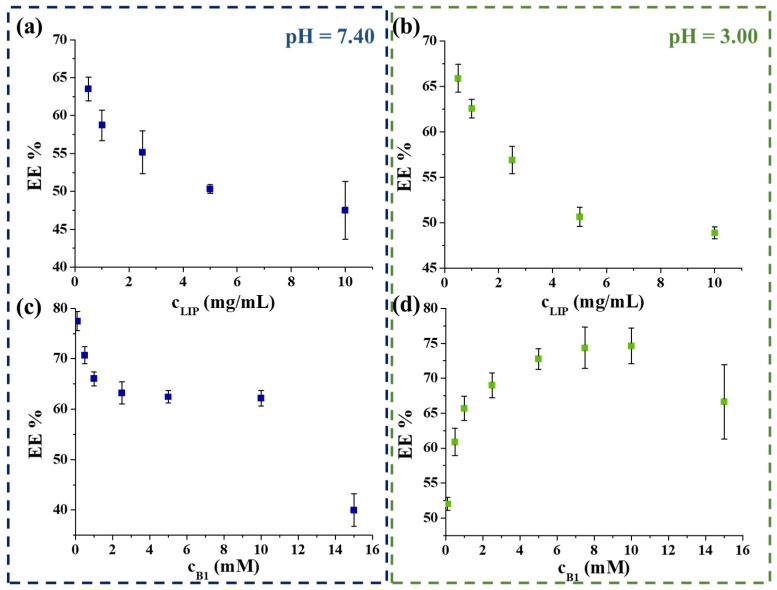
The obtained EE % as a function of the concentration of the applied asolectin (**a**,**b**) and vitamin B_1_ (**c**,**d**) at pH = 7.40 (left) and at pH = 3.00 (right) conditions.

To further optimize the preparation protocol of these vitamin B_1_-containing LIPs, the effects of the amount of the lipid carrier and the vitamin B_1_ were also investigated. During these measurements, the amount of the initial asolectin varied between 0.5–10 mg/mL, while the vitamin B_1_ concentration was constant at 1 mM. As Figure 2 shows, the EE % decreased with the increase of carrier concentration in both cases (Figure 2a,b). The best EE % (63.5 ± 1.8% and 65.8 ± 2.1% in PBS and at acidic medium) was reached by using 0.50 mg/mL carrier content. Next, the maximum amount of the trappable B_1_ was obtained, while the lipid concentration was unchanged at 0.5 mg/mL. During these measurements, individual samples were prepared, where the initial concentration of vitamin B_1_ varied between 0.1–15 mM. Figure 2d clearly shows that the most vitamin B_1_ amount can be encapsulated by applying 10 mM initial vitamin concentration (EE % = 74.6 ± 2.4%) at pH = 3.00. However, at physiological conditions, the increase in the vitamin B_1_ amount results in the decrease in the EE % from ca. 80% to 62.1 ± 1.9% (Figure 2c); the highest vitamin content is achieved in the case of 10 mM vitamin B_1_. As a result, the final encapsulated extent is ca. 6.21 mM and 7.46 mM under physiological and acidic conditions, respectively.

After the optimization of the encapsulation protocols, the structure of the drug-containing carrier systems was characterized. For this purpose, thermoanalytical measurements were carried out, where the formation of the assembling of lipids into LIPs and the relationship between the carrier and drug molecule were identified.

**Figure 3:**
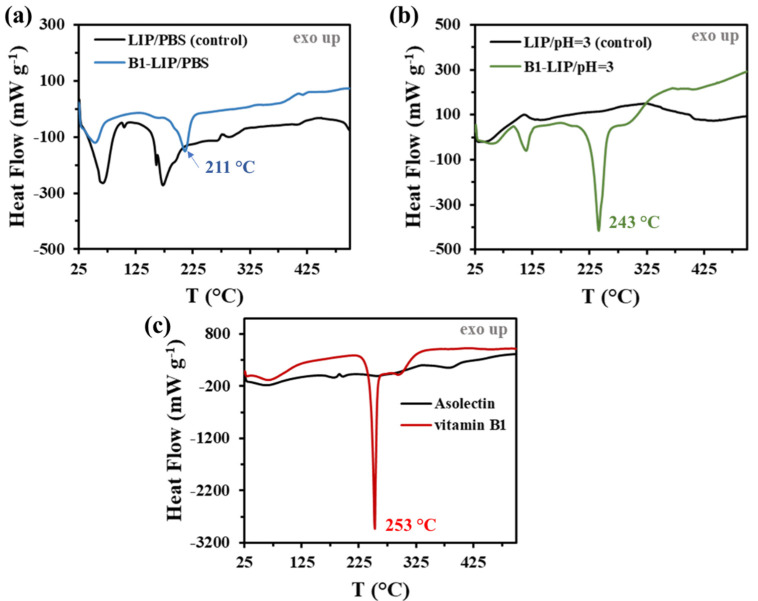
DSC curves of the vitamin-loaded LIPs under (**a**) physiological (pH = 7.4 and 0.15 M NaCl) and (**b**) acidic (pH = 3.0 and 0.20 M KCl) conditions, as well as (**c**) the DSC curves of the pure vitamin B_1_ and the initial asolectin compounds.

Based on the calorimetric and thermogravimetric measurements, the characteristics of the vitamin B_1_-loaded liposomal systems are presented in Figure 3 and Figure S2. The first endothermic peaks on the DSC curves can be related to the evaporation of physically bound water. For vitamin B_1_-containing LIPs, the endothermic peak of the vitamin B_1_ degradation clearly appears, which, depending on the medium, significantly shifts from the T_max_ = 253 °C initial value (Figure 3c) to T_max_ = 211 °C (pH = 7.40; Figure 3a) and T_max_ = 243 °C (pH = 3.00; Figure 3b). The appearance of this peak is clear evidence that vitamin B_1_ is presented in the liposomal system in both cases after purification steps. Taking the heat flow values into account, it can be stated that the higher EE % can be achieved for acidic medium, which is also confirmed by TG measurements (Figure S2). These findings are in good agreement with the previous EE % data. No other heat effect is observed in the DSC curves, thus there is no strong interaction between the carrier and vitamin B_1_, that would significantly affect the drug release.

### 2.2. Analysis of the pH-Dependent Vitamin Release from LIPs

As was mentioned previously, the dissolution of the vitamin B_1_ molecules from the colloidal carriers was studied at two relevant pH conditions (pH = 7.40 and pH = 1.50). Besides our sample, the pure vitamin penetration across the membrane was also investigated as a control and the release profile of a thiamine-hydrochloride-containing tablet which is commercially available in Hungary was also registered.

**Figure 4:**
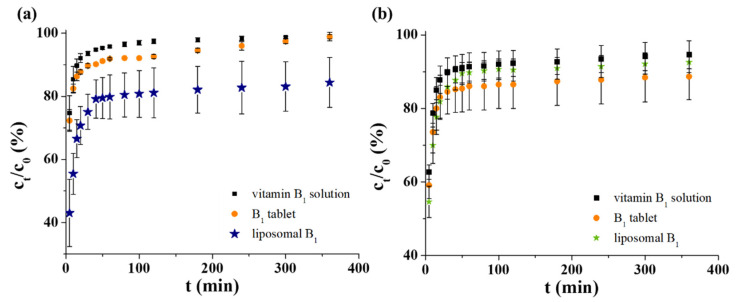
The dissolution profiles of vitamin B_1_ in (**a**) PBS and (**b**) artificial gastric juice.

First, to investigate the possible application as an intravenous drug carrier, the B_1_ release profiles of all three systems were measured in PBS. In all cases, quite a similar starting vitamin content was adjusted (c = 6.0 mM). The registered curves under physiological conditions can be seen in Figure 4a, which clearly shows that 96–97% of the active molecules penetrate through the dialysis membrane after 100 min for pure vitamin solution and ca. 100% is reached at 360 min. It is also observed that ~88–90% of the drug content from the manufactured tablet is released after 1 h. The difference between the pure vitamin solution and the pharmacy formulation has been perhaps influenced by the presence of different excipients. In the case of our sample, it can be estimated that the LIP-based delivery system has the greatest retention for the selected drug: after 60 min., ca. 79% of the total ingredient is dissolved, but at the end of the test time, this value reaches only ca. 84%.

To mimic the gastric condition, an artificial gastric juice was applied, which contains 0.2 M KCl at pH = 1.5. The presence of pepsin was omitted for easier spectrophotometric measurement of samples. For better comparison as well as to avoid the high pH gradient (from pH = 7.4 to pH = 1.5), the manufactured tablets were dissolved in acidic 0.2 M KCl (pH = 3.0) solution. As can be seen in Figure 4b, ~92% of the control non-formulated vitamin molecules diffuse after 100 min and this amount is constant until the end of the examined time frame (360 min). In this acidic medium, the behavior of the tableted and liposomal B_1_ is similar: the overpassed drug content is not changed after 100 min. While the tablet released ca. 91% of the active ingredient, the LIPs left only ~86% vitamin B_1_. Any significant difference between the dissolution curves was not observed. However, considerable drug retention was observed in favor of LIPs in the first 20 min of the release measurements.

**Figure 5:**
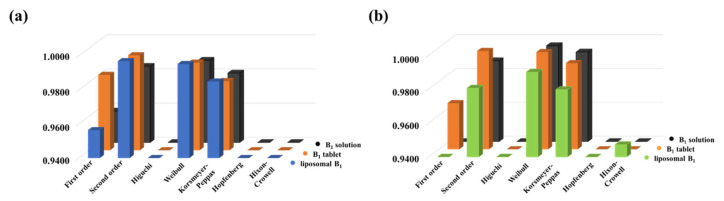
The correlation coefficients (R^2^) for the release curves fitted with various kinetic models under (**a**) physiological and (**b**) acidic conditions.

To analyze the primer release data, the dissolution profiles were fitted by a non-linear technique using seven different kinetic models applying a self-developed and freely available routine [26], which was published previously by our research group. First- and second-order [27,28], Higuchi [29], Weibull [30], Korsmeyer–Peppas [31], Hopfenberg [32] and Hixon-Crowell [20,33] models were tested, where models take different physicochemical parameters into account. The goodness of fit is represented by the correlation coefficients (R^2^), which can be seen in Figure 5, in the case of the PBS (Figure 5a) and acidic (Figure 5b) medium, respectively.

**Figure 6:**
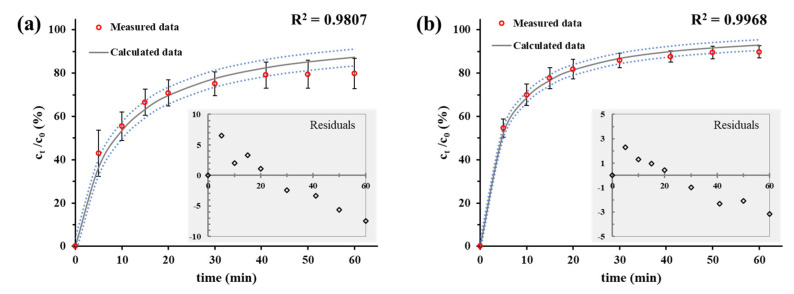
The in vitro dissolution profiles (empty red circles) of liposomal B_1_ with the calculated non-linear second-order kinetic model-based release curves (continuous lines) in (**a**) PBS and (**b**) artificial gastric juice.

The best fit was obtained by using the Weibull model in both cases, but the utilization of the second-order kinetic model provided almost the same tendency. While the Weibull model provides only fitting parameters [30], the half-life (t_1/2_) of the dissolved drug can be determined from the second-order rate equation. The t_d_ value, which is the time after release of 63.2% of drug from the formulation [34], can be determined based on the Weibull model, but it is an empirical model having some deficiencies. On the one hand, there is no kinetic fundament; on the other hand, it has limited use for evaluation in the case of in vivo or in vitro studies. Therefore, the t_1/2_ values are more appropriate for comparison of different formulations. With this in mind, we used the second-order rate equation to evaluate the dissolution curves. As can be seen in Figure 6, the second-order model can provide a narrow confidence interval with good R^2^ in both pHs.

The dotted blue lines represent the confidence intervals, while the gray continuous lines show the predicted dissolution profiles based on the integral second-order kinetic model. This kinetic model provides the rate constant (k) of the dissolution process and the t_1/2_ can be calculated from its values. Based on this parameter, the drug retention of formulated forms can be quantitatively compared with the dissolution feature of the pure active component (drug retention=t12 of formulated formt12 of non−formulated form). These calculated parameters are summarized in Table 1. According to the t_1/2_ values, the drug retention of the different formulations of B_1_ can be estimated. Based on the assessment of the formulation techniques, it can be stated that the LIP-created encapsulation has the largest half-life times under both physiological and acidic conditions. Thus, the longest vitamin retention can be reached by applying a liposomal drug carrier. Namely, the LIP/B_1_ systems have nearly 4.5-fold and 1.5-fold slower drug release compared to the free active ingredient in PBS and artificial gastric juice, respectively.

## 3. Materials and Methods

### 3.1. Materials

For the synthesis of drug-loaded LIPs, asolectin from soybean (25% phosphatidylcholine, Sigma), chloroform (CHCl_3_, 99.9%, Molar), methanol (CH_3_OH, MetOH, 99.9%, Molar), and thiamine hydrochloride (vitamin B_1_, C_12_H_17_ClN_4_OS · HCl, ≥99%, Sigma) were purchased. To adjust the pH, sodium phosphate monobasic monohydrate (NaH_2_PO_4_ × H_2_O; 99%; Sigma), sodium phosphate dibasic dodecahydrate (Na_2_HPO_4_ × 12 H_2_O; 98.5%; Sigma), sodium hydroxide (NaOH, 99.8%, Molar), hydrochloric acid (HCl, 37%, Molar), sodium chloride (NaCl, 99.9%, Molar), and potassium chloride (KCl, 99%, Molar) were applied. Sephadex dextran beads (G50 Medium, Sigma) and Ultrafree^®^ Centrifugal Filter Units (pore size: 0.45 µm, Sigma) were used for the gel filtration to remove the drug excess, while the release profiles were registered by using standard cellulose membrane tubes (cut-off: 12–14 kDa, Sigma). All chemicals were analytical grade and were applied without further purification. The stock solutions were freshly prepared using MQ (Millipore, Milli-Q Integral3) ultrapure water (18.2 MΩ·cm at 25 °C). To compare the release profile of the developed liposome-based nanocarrier to a manufactured system, vitamin B_1_ Zentiva 10 mg (10 mg vitamin B_1_/pill, Zentiva Group, Prague, Czech Republic), as a commercially available dietary supplement, was used.

### 3.2. Methods

#### 3.2.1. Preparation of the B_1_-Loaded Vesicles

Based on the optimized protocol, 100 mg asolectin were dissolved in a 10 mL CHCl_3_:MetOH/9:1 mixture. On the flask wall, the uniform lipid film was evolved by evaporation of the solvent for 15 min at 50 °C. To prepare large unilamellar vesicles, the lipid film was hydrated by magnetic stirring at 900 rpm for 10 min in 200 mL 10 mM vitamin B_1_ solution in PBS or in HCl solution (0.2 M KCl, pH = 3.0) solution depending on further use. The size of the LUVs was controlled by sonication with 37 kHz for 50 min in PBS medium, while the sonication time was 60 min at acidic conditions. The amount of the non-capsulated vitamin B_1_ was removed by centrifuge-assisted gel filtration for 7 min at 7000 rpm.

#### 3.2.2. Characterization Methods

To determine the pH-dependent optical feature of the vitamin B_1_, as well as the drug content and the dissolution profiles, a JASCO V-770 UV-Vis double beam spectrophotometer was applied using a 1 cm quartz cuvette in the range of 200–350 nm. The characteristic absorbance band of vitamin B_1_ was identified at λ_abs_ = 233 nm and λ_abs_ = 246 nm under physiological conditions (PBS buffer, pH = 7.4, 0.15 M NaCl) and artificial gastric juice, respectively. The amount of the encapsulated vitamin B_1_ was determined based on the calibration curves. The encapsulation efficiency (EE %) was calculated by Equation (1):

(1) $$\mathrm{EE}\;\%=\frac{{\mathrm{encapsulated}\;\mathrm{mass}\;\mathrm{of}\;\mathrm{vitamin}\;B}_{1}}{{\mathrm{total}\;\mathrm{mass}\;\mathrm{of}\;\mathrm{vitamin}\;B}_{1}\;\mathrm{in}\;\mathrm{synthesis}}\times 100$$

The measurements of the hydrodynamic diameters (d_H_) and the ζ-potentials were performed on a Malvern Zetasizer NanoZS 4003 apparatus with a He-Ne laser (λ = 633 nm) at 25 ± 0.1 °C and 0.1 M ionic strength. The detection angle was 173°. For the analyses of the stability and the degradation of the drug-loaded and empty LUVs, differential scanning calorimetry (DSC) and thermogravimetry (TG) were applied on a Mettler–Toledo TG/SDTA 851e instrument. The DSC curves were recorded between 25–500 °C with 5 °C/min heating speed and N_2_ gas flow (flow rate: 50 mL/min). During the TG measurements, the observed temperature range was 25–1000 °C, using a 5 °C/min heating rate. The data were analyzed using STARe 12.10 software.

#### 3.2.3. In Vitro Drug Release Measurements

For the in vitro drug release studies, a Hanson Vertical Diffusion Cell (VDC) was used. The VDC has a 61 × 9 mm diameter cylindrical sample holder and a 4 mL sample volume. The drug-loaded LIPs were separated from the release medium by a semipermeable cellulose membrane (cut-off: 12–14 kDa), which was continuously stirred at 800 rpm. The separated buffer flowed through a sample loop with a peristaltic pump, which was connected to a flow-through cuvette of the spectrophotometer. Thus, the absorbance of the dissolved vitamin B_1_ can be registered in a nearly real-time way in PBS and artificial gastric juice [35,36,37]. During the measurements, a thermostatic water bath circulation of VDC was applied at 37 °C and the temperature of the laboratory was fixed at 25 °C. The concentration of the released drug at a specified time can be calculated based on the calibration process (the error of the calculated concentrations was less than ±2%).

#### 3.2.4. Nonlinear Fitting of the Dissolution Profiles

To analyze the registered drug release profiles, the relative concentrations (c_t_/c_0_) of the released vitamin B_1_ as a function of time were calculated. For the mathematical description of the in vitro dissolution, seven different kinetic models were chosen, which are the first- [27] and second-order [28], Higuchi [29], Weibull [30], Korsmeyer–Peppas [31], Hopfenberg [32] and Hixon–Crowell [33,38] models. The calculation was executed based on a previously published open access spreadsheet method [26] by nonlinear fitting. The goodness of the fits was determined by the value of R^2^. Some of the fitted data are presented in Supplementary Materials (Figure S3).

## 4. Conclusions

In this manuscript, vitamin B_1_ molecules were successfully encapsulated into asolectin-based liposomal carrier systems at pH = 7.4 and at acidic conditions. During the synthesis, the sonication time after 10 min magnetic stirring was optimized based on the d_H_ and drug content. The average diameter of the vesicles was ca. 225 and 245 nm in PBS and under acidic conditions, respectively. Besides finding the ideal asolectin (0.5 mg/mL) and initial vitamin B_1_ (10 mM) concentrations, the thiamine-containing LIPs were characterized by thermoanalytical measurements. The TG and DSC clearly showed that the preparation was successful and there are no significant interactions between the vitamin molecules and the carrier lipids. The release curves were evaluated by the non-linear fitting of six different kinetic models. The best fits were determined by the Weibull and second-order kinetic models, which were supported by the correlation coefficients. Based on the second-order kinetics, the t_1/2_ values were calculated and the information about the drug retention was also provided. For the measurements, a commercially available manufactured tablet form of thiamine hydrochloride was chosen. Based on the drug release measurements, it can be stated that the LIP nanocarriers have the best drug retention, which was 4.5-fold and 1.5-fold higher in PBS and acidic medium, respectively, compared to the free and tableted vitamin. To consider the presented results, it can be asserted that the liposomal formulation of the vitamin B molecular family looks promising and is worth considering in the future to develop vitamin-based nanocarriers.

## Figures and Tables

**Table 1:** The half-life (t_1/2_) and the drug retention values determined by second-order kinetic models for different systems.

PBS (pH = 7.4, 0.15 M NaCl)			
	**t_1/2_ (min)**	**t_d_ (min)**	**Drug retention**
pure vitamin B_1_	1.96	2.62	0.00
manufactured B_1_ tablet	2.57	3.49	1.31
liposomal carrier	8.71	10.94	4.44
**Artificial gastric juice (pH = 1.5, 0.2 M KCl)**			
pure vitamin B_1_	2.96	3.11	0.00
manufactured B_1_ tablet	3.93	4.55	1.33
liposomal carrier	4.56	5.91	1.54

## Data Availability

The complete sets of data presented in this study are available on request from the corresponding author.

## Supplementary Materials

The following are available online at www.mdpi.com/article/10.3390/ijms22189851/s1, Figure S1: The d_H_ (black) and ζ-potential (blue) values of the drug-free azolectin-based liposomes depending on the pH. Figure S2: The TG curves of the azolectin, the vitamin B_1_, the vitamin-free and the vitamin-loaded liposomes prepared in PBS and acidic medium. Figure S3: The kinetic evaluations of the LIP/B_1_ systems by non-linear fitting of First-order, Higuchi and Hopfenberg kinetic models.
