# Supplementary material for: The pH-Dependent Controlled Release of Encapsulated Vitamin B1 from Liposomal Nanocarrier
Source: Int J Mol Sci. 2021 Sep 12;22(18):9851. doi: 10.3390/ijms22189851 (PMC8466024; doi:10.3390/ijms22189851)
Supplement: Supplementary file 1 [file ijms-22-09851-s001.zip › ijms-1355993-supplementary.pdf]

SUPPLEMETARY MATERIALS

The pH-dependent controlled release of vitamin B1: encapsulation in liposome and kinetics

Ádám Juhász [1,2], Ditta Ungor [1], Egon Z. Várkonyi [1], Norbert Varga [1] and Edit Csapó [1,2,*]

1 MTA-SZTE "Momentum" Noble Metal Nanostructure Research Group, University of Szeged, Department of Physical Chemistry and Materials Science, H-6720, Rerrich B. sqr. 1, Szeged, Hungary
2 MTA-SZTE Biomimetic Systems Research Group, Department of Medical Chemistry, University of Szeged, H-6720, Dóm sqr. 8, Szeged, Hungary
* Correspondence: juhaszne@chem.u-szeged

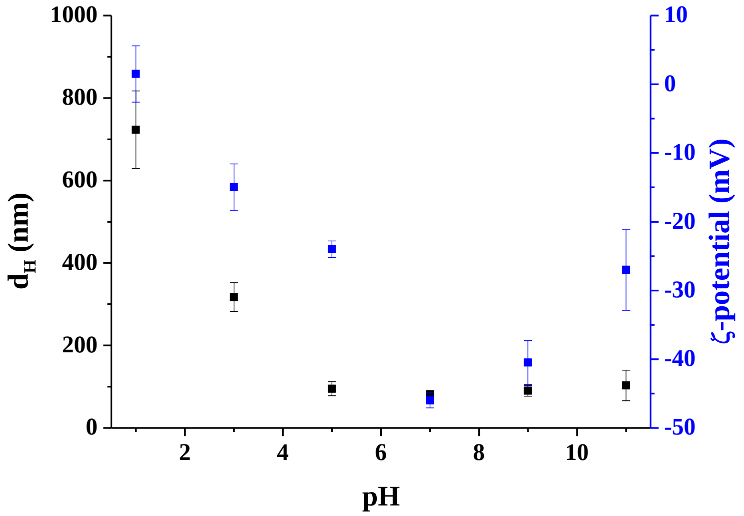

Figure S1. The d_H (black) and ζ-potential (blue) values of the empty azolectin-based liposome depending on the pH.

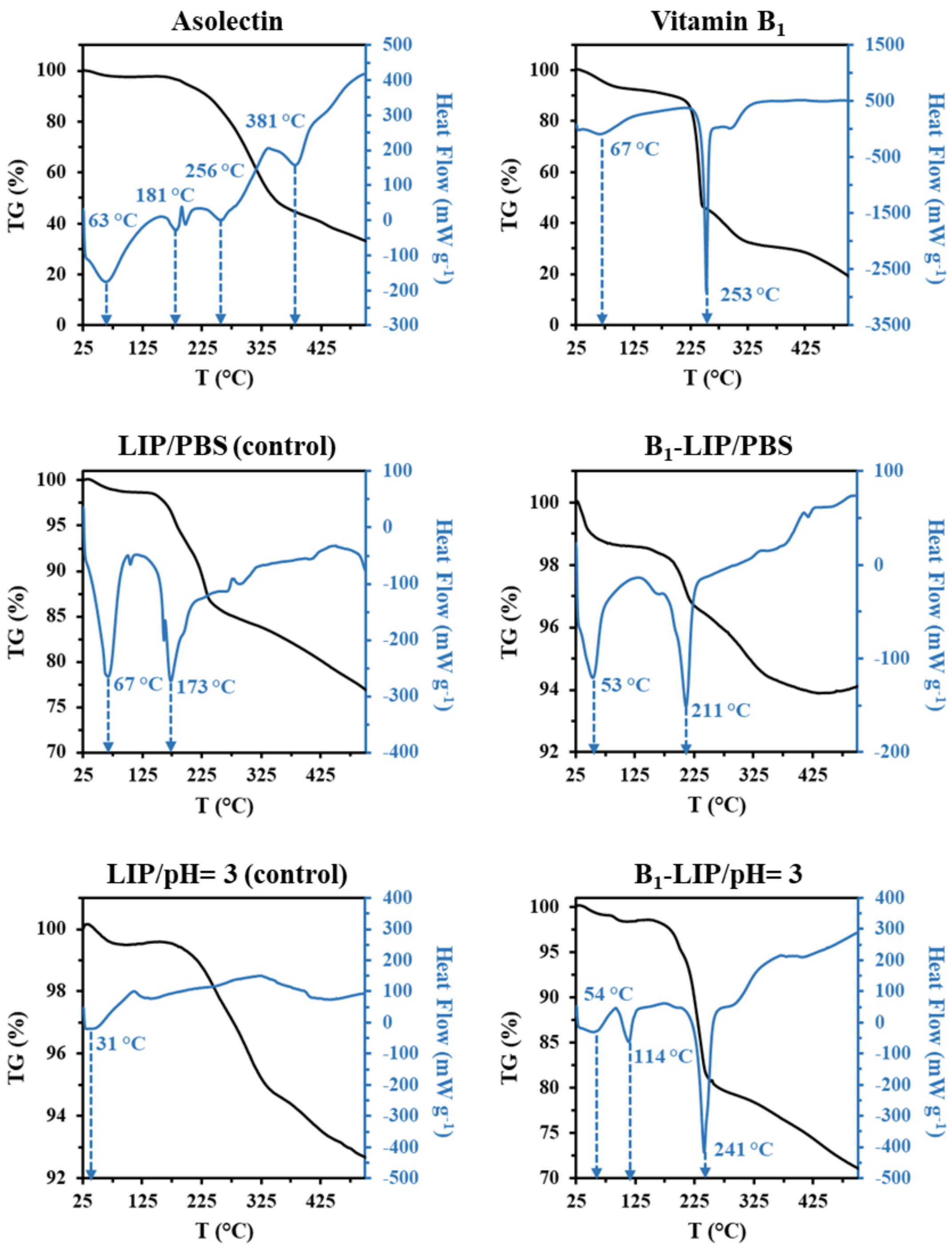

Figure S2. The TG curves of the azolectin, the vitamin B_1, the empty and loaded liposomes prepared in PBS and acidic medium.

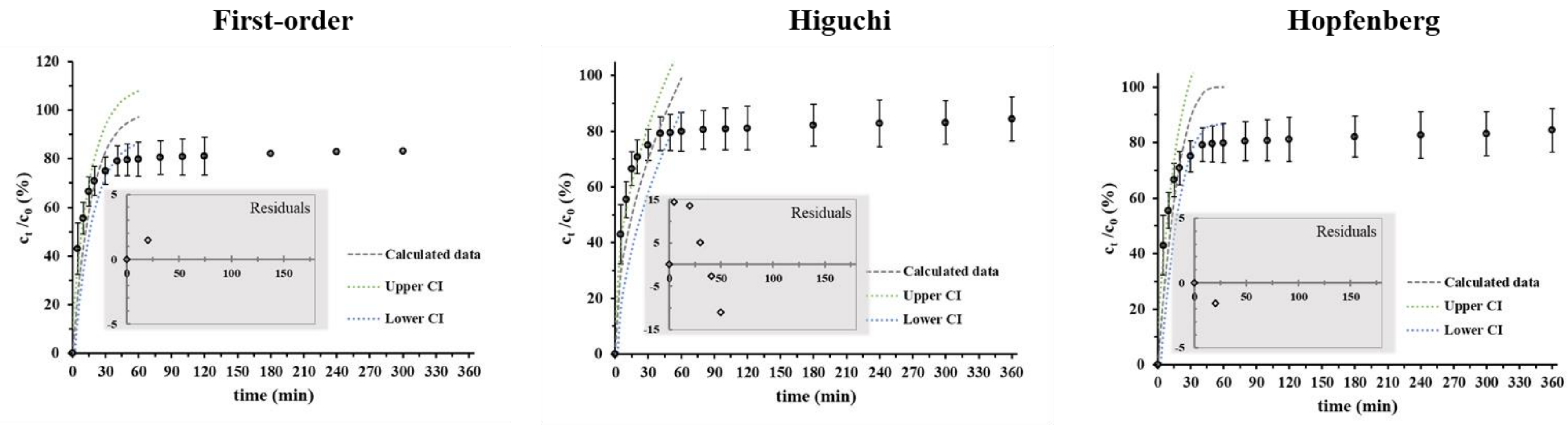

Figure S3. The kinetic evaluations of the LIP/B_1 systems by non-linear fitting of First-order, Higuchi and Hopfenberg kinetic models.
